# Sensorimotor Interaction Against Trauma

**DOI:** 10.3389/fnins.2022.913410

**Published:** 2022-06-14

**Authors:** Giada Persichilli, Joy Grifoni, Marco Pagani, Massimo Bertoli, Eugenia Gianni, Teresa L'Abbate, Luca Cerniglia, Gabriela Bevacqua, Luca Paulon, Franca Tecchio

**Affiliations:** ^1^Laboratory of Electrophysiology for Translational Neuroscience LET'S, Institute of Cognitive Sciences and Technologies ISTC, Consiglio Nazionale Delle Ricerche CNR, Rome, Italy; ^2^Department of Neuroscience, Imaging and Clinical Sciences, University “Gabriele D'Annunzio” of Chieti-Pescara, Chieti, Italy; ^3^Faculty of Psychology, International Telematic University Uninettuno, Rome, Italy; ^4^Unit of Neurology, Neurophysiology, Neurobiology, Department of Medicine, University Campus Bio-Medico of Rome, Rome, Italy; ^5^Studio Psyche Neuroscienze, EMDR Therapist, Rome, Italy; ^6^Independent Researcher, Rome, Italy

**Keywords:** post-traumatic stress disorder (PTSD), visual system, sensorimotor interaction, feedback-synchrony-plasticity (FeeSyCy), eye movement desensitization and reprocessing (EMDR)

## Introduction

**Figure 1:**
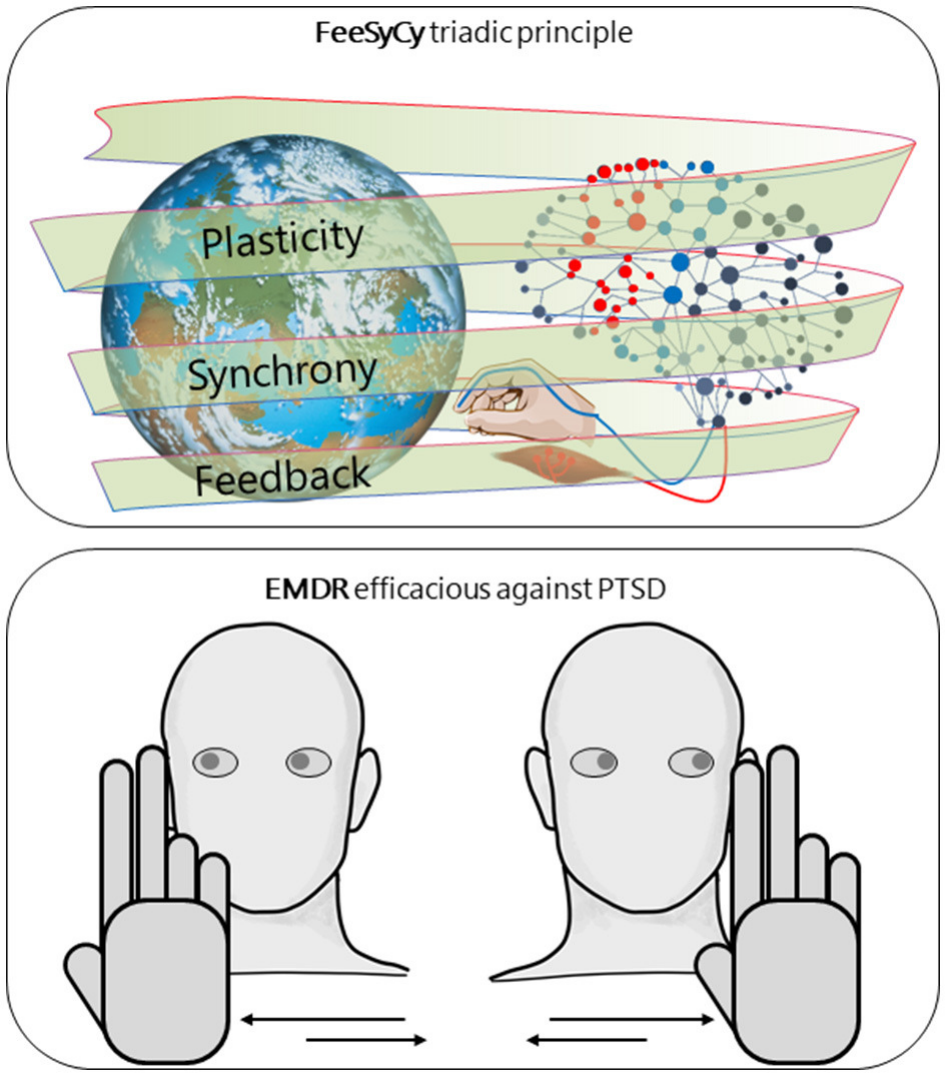
FeeSyCy & EMDR efficacy against trauma. Graphic representation of our central hypothesis, that the triadic principle feedback-synchrony-plasticity (FeeSyCy) governs the adaptive capacity of the body-brain system and underlies the effectiveness of Eye Movement Desensitisation and Reprocessing (EMDR), an elective treatment for post-traumatic stress disorder (PTSD), an illustrative case of reduced adaptive capacity. The neuronal network, shown separately for graphic simplicity, refers to both the patient and the therapist.

Our body-brain system, under physiological conditions, interacts adaptively with the environment. Our central hypothesis is that the triadic principle of feedback-synchrony-plasticity (FeeSyCy, Figure 1) governs the adaptive capacity of the body-brain system. In this opinion, with the perspective goal of contributing to the fight against disorders of major importance, we aim to show how the FeeSyCy principle underlies the efficacy of an elective treatment against an illustrative case of impaired adaptive ability. To this end, we referred to Eye Movement Desensitization and Reprocessing (EMDR) indicated by the World Health Organization (WHO) guidelines (World Health Organization, 2013) as the treatment of choice for post-traumatic stress disorder (PTSD).

## The FeeSyCy Principle

The adaptive body-brain interaction with the environment is essentially rooted in recursive sensorimotor *feedback* loops that compare the expected sensory information coming from the environment due to the enacted behavior and what comes back. The comparison is expressed in the (de) *synchronizations* of the electrical activities of the neuronal involved regions, on whose level depends the *plastic* changes that allow the brain to adapt to ever-changing environmental demands. Thus, the governing principle emerges as the functional triad *feedback-synchrony-plasticity* (FeeSyCy) (Tecchio et al., 2020). This model of individual interplay with the surrounding requirements is in line with the theories of *predictive processing* (Hohwy, 2013), according to which the body-brain system tends to reduce the distance with the target by minimizing the prediction error, resulting from the mismatch between top-down predictions and bottom-up sensory information (Friston and Kiebel, 2009), depending on the delicate modulatory gain control of the involved neuronal population's hierarchies (Kanai et al., 2015). It is becoming increasingly clear how neural networks operate by implementing learning mechanisms that regulate sensory precision according to its functional value that depends on the objective (Grujic et al., 2022).

## PTSD and EMDR

Post-traumatic disorder (PTSD) is one of the possible consequences of a traumatic event that implies the exposition to a threat that may concern the person's integrity and physical condition (Ozer et al., 2003; Vance et al., 2018). PTSD prevalence varies in geographical areas, cultural factors, and different degrees of exposure to traumatic events (Kessler et al., 2017), and occurs twice as often in women than men (Christiansen and Hansen, 2015; Kessler et al., 2017). In this regard, trauma is a relevant example of the need to consider how gender and sex interact with socio-cultural aspects that drive a different manifestation of suffering and access to health care and treatment between men and women (Navarro et al., 2015). Moreover, biological factors underlying sex, such as neuroendocrinology, genomics, and developmental differentiation programmed by gonadal steroids, mediate the individual's impact and outcome of the traumatic event (Sherin and Nemeroff, 2011).

The PTSD provides an exemplificative case of a dramatic alteration of the adaptive physiological nature of the body-brain system. The traumatic experience, when inducing dysregulation of neuronal, hormonal, and immune mechanisms (Ressler et al., 2022) can result in a psychopathological condition characterized by altered adaptive behaviors. According to the Diagnostic and Statistical Manual of Mental Disorders (APA, 2022), criteria to diagnose PTSD include the appearance of intrusive symptoms, avoidance of stimuli associated with trauma, alteration of mood and thinking significant alteration of arousal, and the possible presence of dissociative experiences. At the brain level, evidence shows that neural, neurochemical, and neurobiological alterations induce processing segregation within short-circuits, with structures belonging to the limbic, motor, and sensory systems, storing implicit traumatic memories, and becoming separated from hippocampal-cortical system processing episodic autobiographical memories (Vermetten and Lanius, 2012; Ressler et al., 2022).

Grounding on the theoretical framework of the Adaptive Information Processing (AIP) (Shapiro, 2001), EMDR (Figure 1) consists of a psychotherapy treatment with one session per week typically lasting 50–60 min, comprising 8 phases adapted by the therapist to the patient's situation:

1. Collection of history and treatment plan.
2. Preparation of the patient entering a relationship with the therapist and the identification of a “safe place.”
3. Assessment with identification of TARGET, image, cognition, emotion, feeling, and core of the EMDR treatment. The therapist, in agreement with the patient, stimulates the primary aspects of memory in a safe and structured way to facilitate access to and processing of the most representative or disturbing event. Two scales are assessed: Validity of Positive Cognition (VoC) and Subjective Units of Distress (SUD).
4. Desensitization and reprocessing, stimulating mnemonic activity to allow the TARGET memory to connect to more adaptive processing, involving all associated channels. Treatment is completed with an SUD score 0.
5. Installation, when the patient fully integrates the positive cognition by linking it to the original TARGET event.
6. Body scan: when the body is free from negative sensations, treatment is completed.
7. Closure.
8. Reassessment: verifies the complete processing of the relevant material and collects the SUD and VoC score.

The hallmark of EMDR therapy protocols is the rhythmic bilateral sensorimotor stimulations in phases 4 and 5, typically performed by the patient following with the gaze the index and middle fingers of the therapist, who rhythmically moves them from left to right at a distance of half a meter in front of the patient. EMDR typically requires 12 sessions to resolve the effects of a single trauma.

## FeeSyCy Principle and EMDR Efficacy Mechanisms

The visual system works *par excellence* governed by the FeeSyCy principle, with motor control being the key to sensory perception (Harris and Wolpert, 1998), as the observation of an even static image, we enact ocular motor programs guided by cognitive-emotional representations developed throughout our lives (Yarbus, 1967). Notably, eye movements are essential, if the ocular saccades are blocked, we are perceptually blind (Alexander and Martinez-Conde, 2019). Thus, to target and unlock brain circuits triggered by traumatic events, EMDR utilizes the most powerful tool of sensorimotor interaction, namely vision.

The strategic function of the gaze for human life is evident when recalling its role in establishing the relationship at the origin of survival, the one between the infant and their mother (Stern, 1990; Winnicot, 2005). The gaze creates an expressive form of communication—long before the baby has access to speech or intentional action, and sustains a pattern of social interactions essential for his/her future relationships (Bowlby, 1958). The role of the gaze can also be recognized in the physiological hierarchy being the last function to be lost, to the extent that even in locked-in patients gaze remains under active control despite the loss of all other motor functions (Yang et al., 1989).

In deepening the important role of bilateral eye movements in the EMDR treatment, we consider here a study that compared the efficacy against PTSD of three procedures (Sack et al., 2016). People either fixed their gaze on the hand of the therapist while it was moving (eye movements), or while it was not (fixed gaze) or had no specific focus (absence of focus). The two conditions of eye movements and fixed gaze both improved PTSD symptoms compared to the absence of focus. Moreover, bilateral rhythmic stimulation (BRHYS) induced by eye movements has higher efficacy than auditory BRHYS (van den Hout et al., 2012).

Plastic adaptive abilities subtending reacquired equilibration of intracerebral functional connectivity in reaction to trauma (Santarnecchi et al., 2019) require a re-balance of the excitatory-inhibitory mechanisms that at the cortical level mediate the acquisition of new skills (Wehr and Zador, 2003; Falkner et al., 2010; Kolasinski et al., 2017), as well as the balance between hemispheric homologs (Meyer et al., 1998). EMDR–typical bilateral rhythmic procedures that are likely to facilitate this re-balancing emergence, enabling a neurobiological condition capable of promoting the integration of traumatic memories at the cortical level (Pagani et al., 2007, 2012; Landin-Romero et al., 2018).

We describe below three main mechanisms indicated as underlying the EMDR procedure, activated by the eyes' rhythmic bilateral sensorimotor stimulations in the Desensitization and Installation phases.

### The Orienting Reflex Mechanism

The orienting reflex is a natural response to new environmental stimuli (Donchin, 1981), increasing readiness to respond to danger (Vanderwolf, 1969; Korte et al., 2005). It is typically triggered by sight or hearing and induces the shift of spatial attention in the direction of the new stimulus. Brief alarming stimuli evoke an immediate orienting response, typically characterized by cutaneous vasoconstriction and respiratory activation, which increases sympathetic activity. In the absence of danger, the reaction to novelty, rather than activating the fear response—expressed in the fight, flight, or freeze—relaxes the system. By strengthening the psychotherapist's interaction with the patient within the therapeutic relationship, the BRHYS, typical of the EMDR strategy, supports the reprocessing of the fear response generated by the traumatic memory (de Voogd et al., 2018). In agreement with the FeeSyCy principle, key feedback processing is reactivated within nuclei of the brainstem, cerebral cortex, and regions involved in the hypothalamic-pituitary-adrenal axis (Lupien and McEwen, 1997; de Voogd et al., 2018).

### The Working Memory Mechanism

Working memory is crucial for information storage and has a task- and time-related limited capacity. When involved in the BRHYS-mediated interaction of the EMDR session during the recall of traumatic memories, the working memory retrieves the traumatic information without the same vividness and this leads to an emotional detachment from the traumatic material (Van Den Hout et al., 2011). In terms of the FeeSyCy principle, in prefrontal cortical neuronal populations, the neural hallmark of working memory, synchronizations induced by bilateral rhythmic stimulations, saturate reverberating synaptic excitatory neuronal activation by inhibiting the delay period activity that supports working memory (Wang, 1999; Grossberg, 2013).

### The Slow-Wave Sleep Mechanism

Under normal conditions, during the waking state, information with emotional and episodic values is transferred to the amygdala and hippocampus, respectively, where it is temporarily retained. Among the complex phenomena that sustain memory consolidation during sleep (Klinzing et al., 2019), during the slow-wave sleep (SWS) phase, the short-term memories are transferred back to the neo-cortex where the reverberant activities of thalamic origin favor the epigenetic modifications of neuronal dendrites and synapses that represent the long-term memory engram of this information (Ribeiro et al., 2007; Almeida-Filho et al., 2018). Concurrent to the neo-cortex modifications, amygdala and hippocampus synapses undergo a depotentiation. Upon extreme stress situations, like in PTSD, a maximal potentiation of the amygdala synapses occurs, causing a sort of local short-circuit that prevents the transfer of the emotional memory traced to the cortical areas where integration with the episodic memories coming from the hippocampus takes place. Meaning, the emotional traumatic memory cannot be merged with its autobiographical episodic memory trace (Harper et al., 2009). According to the SWS hypothesis of EMDR, the rhythmic eye movements, while following the therapist's fingers concomitant with the traumatic episodic, recall reproduces the SWS stimulations, reactivating the integration process (Pagani et al., 2017). In terms of the FeeSyCy principle, a systemic feedback mechanism synchronizes circadian control throughout the body, where each cell—with its rhythmicity—in turn, depends on metabolic regulation as a result of nutrient, energy, and redox levels signal sent to the cellular “clock” to reinforce circadian rhythmicity and adapt physiology to tissue-specific time demands (Reinke and Asher, 2019). In the brain, the cortico-thalamic cycles that regulate neuronal activity in support of sleep plasticity have a feedback structure (Contreras et al., 1996).

## Discussion

What is expressed here (Figure 1) suggests that, in clinical practice, the EMDR therapist can enhance the Assessment, Desensitization and Reprocessing, and Installation phases by favoring the choice of multisensory inputs with deep evocative power (Silva et al., 2015; Brillantes-Evangelista, 2016; Matthijssen et al., 2017; Gerge et al., 2019) and high level of gratification (Bee et al., 2008; Van Ommeren, 2013; Berliner et al., 2019). The contents of the stimulation will be chosen together with the therapist by the patient according to her/his preferences. These personalized sensorimotor interactions enter, along the same lines drawn by personalized neuromodulation interventions inspired by the FeeSyCy model (Tecchio et al., 2014; Cottone et al., 2017, 2018; Cancelli et al., 2018; Armonaite et al., 2021), in the global approach of precision medicine informed by the individual bio-psycho-social history (Collins and Varmus, 2015).

## Author Contributions

GP and FT drafted the paper. MP deepened the EMDR mechanism. GB deepened the EMDR clinical protocol descriptions. LC and MB integrated the clinical perspective. LP contributed in the service development ideas. JG, EG, and TL'A shared the neurophysiology-psychotherapy connection. All authors contributed to the final writing, contributed to the article, and approved the submitted version.

## Funding

This study was supported by the POR FESR LAZIO 2014-2020 (Lazio Innova on behalf of Regione Lazio, Gecoweb A0320-2019-28109, cup B89C20001430002), Project n. 28109, Title: Digital Helpers per e-Communities in Sanitá, Acronym: DHelp4H.

## Conflict of Interest

The authors declare that the research was conducted in the absence of any commercial or financial relationships that could be construed as a potential conflict of interest.

## Publisher's Note

All claims expressed in this article are solely those of the authors and do not necessarily represent those of their affiliated organizations, or those of the publisher, the editors and the reviewers. Any product that may be evaluated in this article, or claim that may be made by its manufacturer, is not guaranteed or endorsed by the publisher.
